# Survival outcomes among hospitalized patients with dementia: a propensity score matching analysis

**DOI:** 10.1007/s13760-025-02746-7

**Published:** 2025-03-15

**Authors:** Henry Oliveros Rodríguez, Natalia Diaz-Dussan, Yahira Guzmán-Sabogal, Juliana Proaños, Eduardo Tuta-Quintero

**Affiliations:** 1 School of Medicine, Universidad de La Sabana, Autonorte de Bogota Km 7, La Caro, Chía, Colombia; 2 Master’s Candidate in Epidemiology, Universidad de La Sabana, Chía, Colombia

**Keywords:** Dementia, Hospitalization, Survival analysis, Mortality, Comorbidity

## Abstract

**Background:**

Hospitalized patients with dementia exhibit high mortality rates, underscoring the importance of investigating variables associated with reduced survival. This study aims to determine the incidence of dementia among hospitalized patients and survival rates at 1 and 3 years post-hospitalization.

**Methods:**

A retrospective cohort study was conducted using administrative databases from the Ministry of Health of Colombia. One- and three-year survival rates, along with adjusted hazard ratios for survival accounting for comorbidities included in the Charlson Index, were assessed using a Cox proportional hazards model. This analysis was performed for patients with dementia versus a control group without dementia. Additionally, findings were compared with those from an inverse propensity score weighting model.

**Results:**

6.769 (1.04%) patients were diagnosed with dementia, and 5798 (85.65%) were over 65 years of age. The unadjusted HR, the HR adjusted using the proportional hazards Cox model, and the HR obtained through propensity score matching (PSM) were 10.32 (95% CI 9.82 to 10.84), 1.69 (95% CI 1.60 to 1.78), and 1.32 (95% CI 1.02 to 1.71), respectively. The 1-year adjusted mortality rates for patients with dementia and those without were 12.5% and 1.31%, respectively, while the corresponding 3-year adjusted mortality rates were 21.25% and 2.76%. Through PSM, we determined that the mean survival time for patients with dementia, in comparison to those without, was − 0.98 months (95% CI: -0.65 to -1.94; *p* < 0.001).

**Conclusions:**

Dementia significantly reduces survival rates of hospitalized patients, regardless of other comorbidities. Specifically, our research revealed that dementia was associated with a decrease in 3-year survival by an average of 0.98 months.

**Supplementary Information:**

The online version contains supplementary material available at 10.1007/s13760-025-02746-7.

## Introduction

Dementia is a chronic, acquired, and progressive disease that leads to a gradual loss of cognitive function and eventual mortality [1, 2]. Furthermore, it is a heterogeneous pathology with multiple etiologies, typically diagnosed in advanced stages [3]. This results in a high mortality rate linked to comorbidities in affected patients [4]. Numerous studies, employing diverse methodologies and diagnostic criteria, have consistently shown an association between dementia, and decreased short- and long-term survival (60–90% annual survival rate) [5–8]. Mortality is influenced by several factors, including advanced age, male sex, decreased functional status (> 6 months), and medical comorbidities such as type 2 diabetes mellitus, cardiovascular diseases, or malignancies. Additionally, the frequency of hospital admissions in these patients is associated with severe cognitive dysfunction, polypharmacy, cachexia, dysphagia, and malnutrition [7, 9–12].

Dementia poses a growing public health challenge, carrying substantial social and economic ramifications [13, 14]. According to recent estimates from the Alzheimer’s Association, the total direct medical expenses associated with various types of dementia are projected to surge from $236 billion in 2016 to over $1 trillion in the United States by 2050 [15]. This escalation is attributed to an increased prevalence of dementia, prolonged life expectancy, and a high prevalence of comorbidities and caregiving responsibilities [16, 17].

In Colombia, the National Survey of Health, Well-being, and Aging (SABE) reported a dementia prevalence of 9.4% (95% CI: 7.7–11.4) in 2015, with a positive correlation to age and reaching a prevalence of 57.4% in individuals aged > 85 years [13, 17, 18]. Despite this, there is a scarcity of studies in Colombia assessing the prognosis of patients diagnosed with any type of dementia concerning mortality. Therefore, this study aimed to identify cases of dementia diagnosed for the first time in 2016, ensuring that they had not received this diagnosis in the previous two years, as well as to determine their survival rates at one and three years post-hospitalization.

## Methods

A retrospective cohort study was conducted using administrative data from the Integrated Social Protection Information System (SISPRO) of the Colombian Ministry of Health. The study cohort included patients over 18 years of age who were hospitalized for any cause during the analysis period. To capture a comprehensive description of health conditions, comorbidities were identified using the Charlson comorbidity index, which includes dementia among its categories. Propensity score matching (PSM) was performed to balance baseline differences between patients with dementia and those without a dementia diagnosis.

### Information sources

The SISPRO database contains information on hospitalizations provided by health insurance companies within the Colombian healthcare system. This database includes unique codes for procedures performed (CUPS), service dates, age, sex, insurance information, municipality details, ICD-10 codes, and the cost incurred for each episode of care (***Supplementary Table 1***). Additionally, we utilized a second database consisting of death certificates, extracting details such as date of death and diagnoses associated with the cause of death from the Single Registry of Affiliates (RUAF). We used ICD-10 codes to identify patients diagnosed with dementia in the SISPRO database, creating a cohort of patients with and without dementia. Subsequently, we used RUAF data to identify deceased patients matching those in the cohort, applying administrative censoring up to December 31, 2018.

## Population

We determined a sample size of 456 patients through the formula described in Supplementary Table 2, considering the comparison between time and events. However, to accommodate subgroup analysis involving treated patients with dementia, we opted for an increased sample size. From a pool of 3,850,772 hospitalized patients, we randomly selected 660,000 individuals aged over 18 years, admitted in Colombia between January 1st, 2016, and December 31st, 2016. In cases involving multiple hospitalizations, we selected the first admission and excluded 8234 patients (1.24%) due to incomplete data.

## Study variables

The exposure variable in this study was the dementia diagnosis, while the primary outcome focused on mortality from any cause over an average follow-up period of 3 years. Charlson Index comorbidities were computed based on data gathered for two years preceding hospitalization for each patient, spanning 2014 and 2015 (Supplementary Fig. 1). We established algorithms to accurately identify each comorbidity, incorporating ICD-10 codes, CUPS (Supplementary Table 1). Mortality records were integrated with the records of hospitalized patients through an identification number.

For this study, we defined incident cases of dementia as patients who did not have a prior diagnosis of dementia recorded in the administrative database during the two years preceding the index event (Supplementary Fig. 1). This definition is based on the review of available clinical records documenting diagnoses established by healthcare professionals in the context of hospitalizations or prior medical care included in the database.

### Statistical analysis

Between-group comparisons of baseline characteristics utilized standardized differences for each variable. To mitigate potential selection biases arising from a lack of randomization and to adjust for confounding variables, we employed matching techniques based on the propensity index, following Austin’s recommendations [19, 20]. Hazard ratios (HR) were calculated with administrative censorship as of December 31st, 2018, resulting in an average follow-up time of three years.

For PSM, we obtained the propensity score through a logistic regression model incorporating covariates such as the 16 comorbidities outlined in the Charlson index, age, sex, and country regions. The closest neighbor algorithms with a 1:1–1:5 ratio, caliper method, Kernel, and inverse probability weighting (IPW) were utilized in PSM. Criteria for assessing optimal between-group balance of baseline characteristics included standardized differences of < 0.1 and a Rubin index of < 25% [21]. Subsequently, HR were calculated for both paired groups to obtain unbiased estimates, and 95% confidence intervals were determined by estimating robust standard errors.

Survival analysis involved describing the likelihood of survival from hospital admission using Kaplan-Meier survival curves. Differences in survival versus exposure time were assessed using the log-rank test. HR and corresponding 95% confidence intervals (CI) were calculated, adjusting for covariates using the Cox proportional model. Proportional hazards assumptions were verified, and variables were included based on their significance and biological plausibility (Supplementary Fig. 2).

Statistical significance was set at 0.05. Statistical analyses were conducted using STATA 15.0 (StataCorp. 2017. *Stata Statistical Software: Release 15*. College Station, TX: StataCorp LLC.)

## Results

### Descriptive analysis

**Fig. 1:**
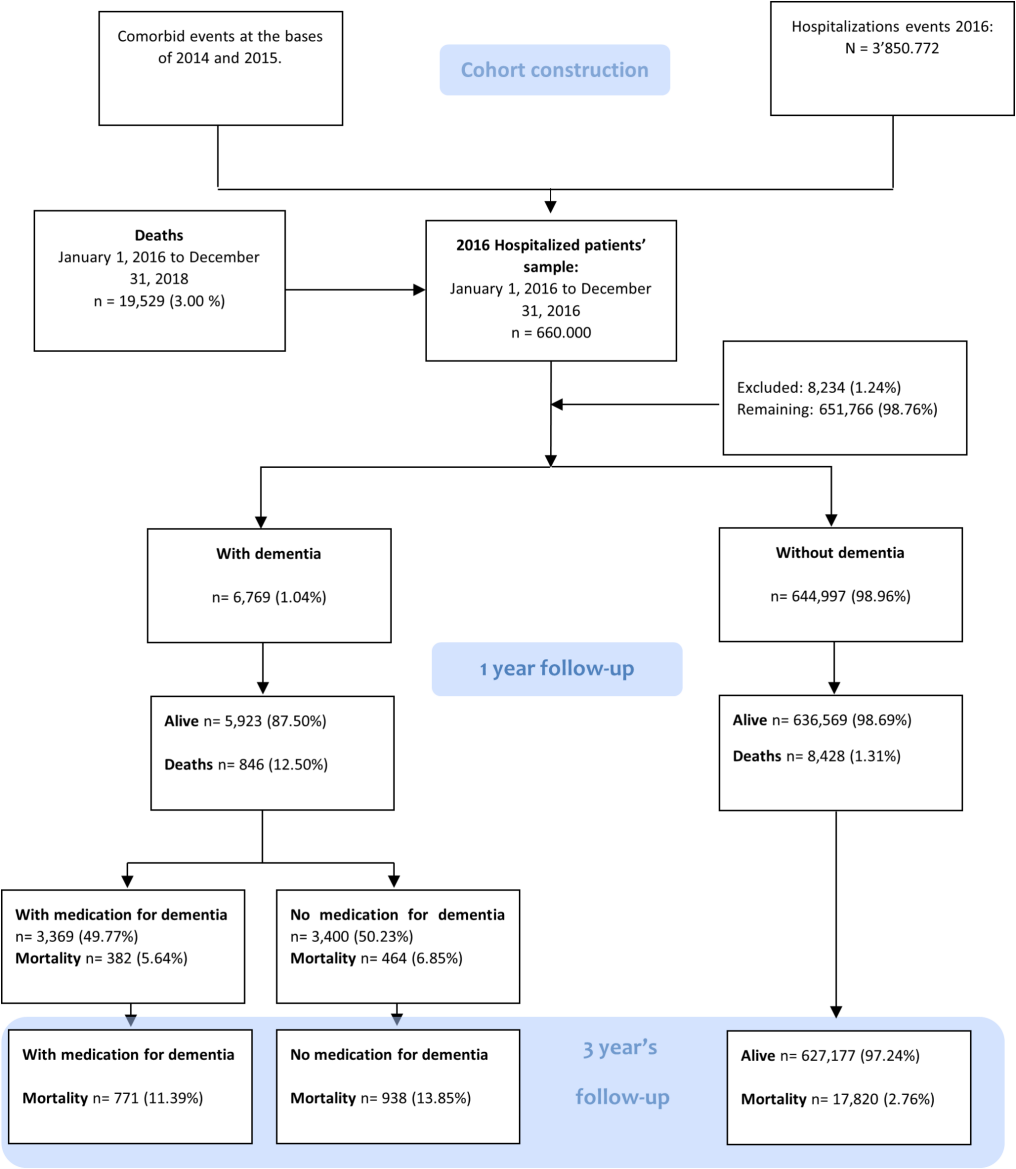
Flow chart of the included patients

Between January 1, 2016, and December 31, 2016, a total of 3,850,772 patients underwent hospitalization in the Colombian territory. We randomly selected a sample of 660,000 patients, excluding 8234 (1.24%) due to incomplete data. Ultimately, our study comprised 651,766 patients, with 6769 (1.04%) diagnosed with dementia. Among those diagnosed with dementia, 5798 (85.65%) were aged over 65 years, and records of medication use were available for 3369 patients (49.77%) (Fig. 1). Among the 6769 patients with dementia, there were 3369 (50%) patients who received anti-dementia medications, with rivastigmine accounting for 2359 (70%) of the formulation records while amantadine and memantine accounted for the remaining 1010 (30%). There were no records of other anti-dementia drugs.

Among patients diagnosed with dementia, the most frequently observed comorbidities included diabetes mellitus with/without complications (36.84% and 35.15%, respectively), chronic obstructive pulmonary disease (34.04%), and stroke (19.87%). Conversely, in patients without dementia, the prevalent comorbidities were diabetes mellitus with/without complications (10.42% and 10.24%, respectively), chronic obstructive pulmonary disease (9.52%), and peptic ulcer (5.57%) (Table 1).

**Table 1:** General characteristics of hospitalized patients during 2016

Baseline characteristics	Exposure status					
	Full sample		With Dementia	Without Dementia		Standardized differences
	*n =* 651,766		*n =* 6,769	*n =* 644,997		
*Age mean*	44.15		77.00	43.80		20.41
*Age n (%)*						
18–50	418,503 (64.21)		351 (5.19)	418,152 (64.83)		−160.2
51–60	86,712 (13.30)		325 (4.80)	86,387 (13.39)		−30.2
61–70	65,054 (9.98)		781 (11.54)	64,273 (9.96)		5.1
61–70	47,148 (7.23)		1,755 (25.93)	45,393 (7.04)		52.6
71–80	28,705 (4.40)		2,729 (40.32)	25,976 (4.03)		97.1
81–90	5,551 (0.85)		818 (12.08)	4,733 (0.73)		47.6
91–100	93 (0.01)		10 (0.15)	83 (0.01)		4.8
*Region, n (%)*						
Atlantic	120,889 (18.55)		1,187 (17.54)	119,702 (18.55)		−2.7
Bogotá	191,099 (29.32)		1,675 (24.75)	189,424 (29.32)		−10.4
Central	145,450 (22.32)		1,873 (27.67)	143,577 (22.32)		12.5
Oriental	102,031 (15.65)		1,007 (14.88)	101,024 (15.65)		−2.2
Pacific	86,856 (13.33)		1,013 (14.97)	85,843 (13.33)		4.8
Others state	5,441 (0.83)		14 (0.21)	5,427 (0.83)		−8.8
*Gender, n (%)*						
Female sex	408,897 (62.74)		4,353 (64.31)	404,544 (62.72)		−3.3
Male sex	242,869 (37.26)		2,416 (35.69)	240,453 (37.28)		−3.3
*Comorbidities, n (%)*						
Myocardial Infarction	16,598 (2.55)		663 (9.79)	15,935 (2.47)		30.9
Congestive heart failure	5,791 (0.89)		266 (3.93)	5,525 (0.86)		20.2
Peripheral vascular disease	6,073 (0.93)		254 (3.75)	5,819 (0.90)		19.0
Cerebrovascular disease	10,878 (1.67)		1,345 (19.87)	9,533 (1.48)		62.4
Chronic pulmonary disease	63,709 (9.77)		2,304 (34.04)	61,405 (9.52)		62.2
Connective tissue disease	20,359 (3.12)		418 (6.18)	19,941 (3.09)		14.7
Ulcer disease	36,769 (5.64)		835 (12.34)	35,934 (5.57)		23.9
Mild liver disease	1,582 (0.24)		50 (0.74)	1,532 (0.24)		7.2
Severe liver disease	806 (0.12)		21 (0.31)	785 (0.12)		4.1
Diabetes mellitus	68,457 (10.50)		2,379 (35.15)	66,078 (10.24)		62.3
Diabetes mellitus with complications	69,703 (10.69)		2,494 (36.84)	67,209 (10.42)		65.4
Metastatic solid tumor	6,231 (0.96)		163 (2.41)	6,068 (0.94)		11.5
AIDS	5,658 (0.87)		427 (6.31)	5,231 (0.81)		30.0
Any tumor	1,988 (0.31)		43 (0.64)	1,945 (0.30)		4.9
Plegia	516 (0.08)		34 (0.50)	482 (0.07)		8.0
Kidney failure	6,512 (1.00)		164 (2.42)	6,348 (0.98)		11.1
*Mortality, n (%)*						
One year mortality	9,274 (1.42)		846 (12.5)	8,428 (1.31)		
Three year mortality	19,529 (3.00)		1,709 (21.25)	17,820 (2.76)		

CI, confidence interval; AIDS: acquired immunodeficiency syndrome

## Effect of dementia on survival

The 1-year mortality rates for all patients, patients with dementia, and patients without dementia stood at 1.42%, 12.5%, and 1.31%, respectively. Correspondingly, the 3-year mortality rates were 3.0%, 21.25%, and 2.76%, respectively, as outlined in Table 1, along with standardized differences.

The mean 1-year survival times for patients with dementia and those without were 23.5 months (95% CI 23.48–23.6) and 23.9 months (95% CI 23.96–23.96), respectively. Likewise, the corresponding mean 3-year survival times were 31.78 months (95% CI 31.59–31.97) and 35.54 months (95% CI 35.53–35.54).

## Balance of base characteristics by PSM

Table 2 presents the fundamental characteristics of both patient groups post PSM, which facilitated a well-balanced comparison using the closest neighbor algorithm in a 1:1 ratio, yielding a Rubin index of 4.6 (Supplementary Fig. 3). Following pairing, the disparity in mean survival time between groups was − 0.98 months (95% CI −0.65 to −1.94; *P* < 0.001), comparing patients with dementia to those without.

**Table 2:** General characteristics of patients hospitalized during 2016 before and after matching

	Without matching			With matching		
Baseline characteristics	With Dementia	Without Dementia	Standardized differences	With Dementia	Without Dementia	Standardized differences
*Age*						
18–50	0.051	0.648	−160.2	0.051	0.051	0.1
51–60	0.048	0.133	−30.2	0.048	0.046	0.5
61–70	0.115	0.099	5.1	0.115	0.114	0.4
61–70	0.259	0.070	52.6	0.259	0.265	−1.6
71–80	0.403	0.040	97.1	0.403	0.403	−0.1
81–90	0.120	0.007	47.6	0.120	0.117	1.2
91–100	0.001	0.000	4.8	0.001	0.001	−1.0
*Region*						
Atlantic	0.175	0.185	−0.27	0.175	0.172	0.7
Bogotá	0.247	0.293	−10.4	0.247	0.245	0.5
Central	0.276	0.222	12.5	0.276	0.277	−0.1
Oriental	0.148	0.156	−2.2	0.148	0.152	−1.2
Pacific	0.148	0.133	4.8	0.148	0.150	−0.1
Others state	0.002	0.008	−8.8	0.002	0.001	0.2
*Gender*						
Female sex	0.356	0.372	−3.3	0.356	0.355	0.3
*Comorbidities*						
Myocardial Infarction	0.097	0.024	30.9	0.097	0.096	0.5
Congestive heart failure	0.039	0.008	20.2	0.039	0.036	1.9
Peripheral vascular disease	0.037	0.009	19.0	0.037	0.034	2.0
Cerebrovascular disease	0.198	0.014	62.4	0.198	0.198	0.2
Chronic pulmonary disease	0.340	0.095	62.2	0.340	0.339	0.3
Connective tissue disease	0.061	0.030	14.7	0.061	0.059	1.3
Ulcer disease	0.123	0.055	23.9	0.123	0.124	−0.4
Mild liver disease	0.007	0.002	7.2	0.007	0.007	−0.2
Severe liver disease	0.003	0.001	4.1	0.003	0.002	1.6
Diabetes mellitus	0.351	0.102	62.3	0.351	0.354	−0.8
Diabetes mellitus with complications	0.368	0.104	65.4	0.368	0.368	−0.0
Metastatic solid tumor	0.024	0.009	11.5	0.024	0.020	2.5
AIDS	0.063	0.008	30.0	0.063	0.061	0.6
Any tumor	0.006	0.003	4.9	0.006	0.006	0.0
Plegia	0.005	0.000	8.0	0.005	0.004	0.3
Kidney failure	0.024	0.009	11.1	0.024	0.023	0.5

AIDS: acquired immunodeficiency syndrome

### Cox proportional risk model

The unadjusted HR, the HR adjusted using the proportional hazards Cox model, and the HR obtained through PSM were 10.32 (95% CI 9.82 to 10.84), 1.69 (95% CI 1.60 to 1.78), and 1.32 (95% CI 1.02 to 1.71), respectively (Table 3Supplementary Fig 4).

**Table 3:** Survival hazard ratio

Time frame	HR unadjusted *(95% CI)	HR adjusted * (95% CI)
1-year survival for patients with dementia	10.32 (9.82 to 10.84)	1.69 (1.60 to 1.78)

HR, Hazard ratio; CI, confidence interval.

*Adjusted for age, sex, Charlson comorbidity index and regions of Colombia by COX model.

**Fig. 2:**
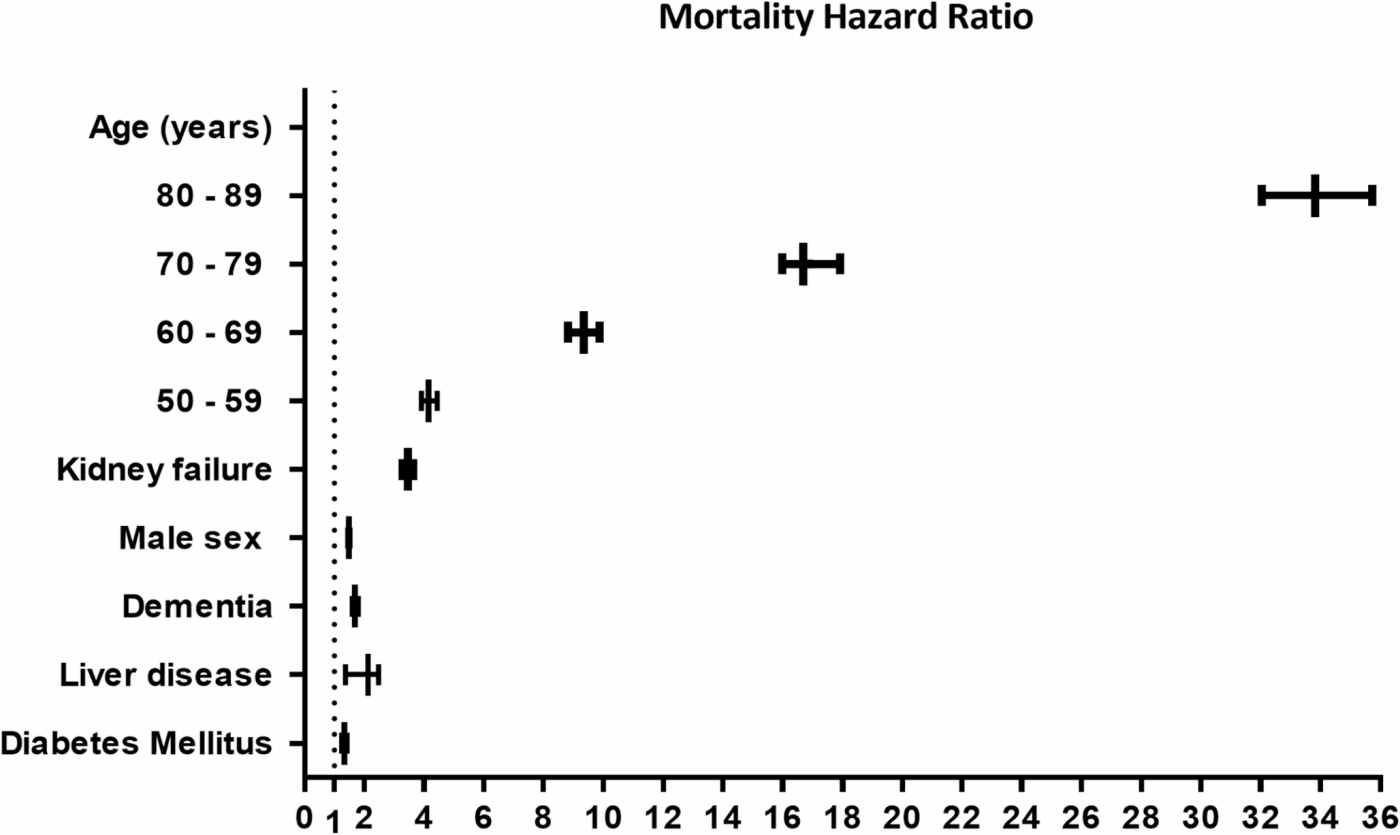
Three-year mortality hazard ratio for dementia with adjustment for sex, kidney failure, liver disease, diabetes mellitus, and age categories

**Fig. 3:**
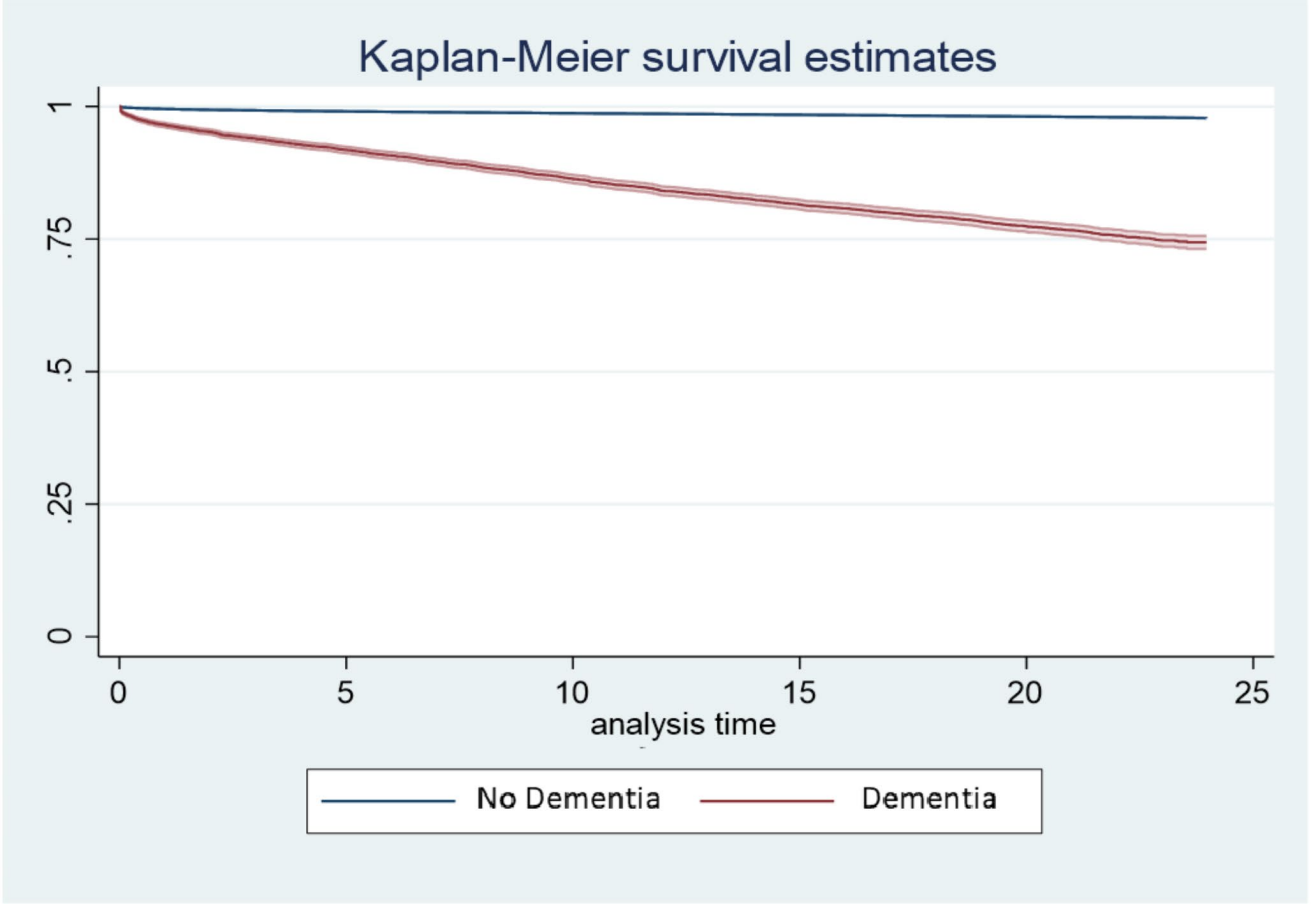
Three-year survival curves not adjusted for confounding variables

**Fig. 4:**
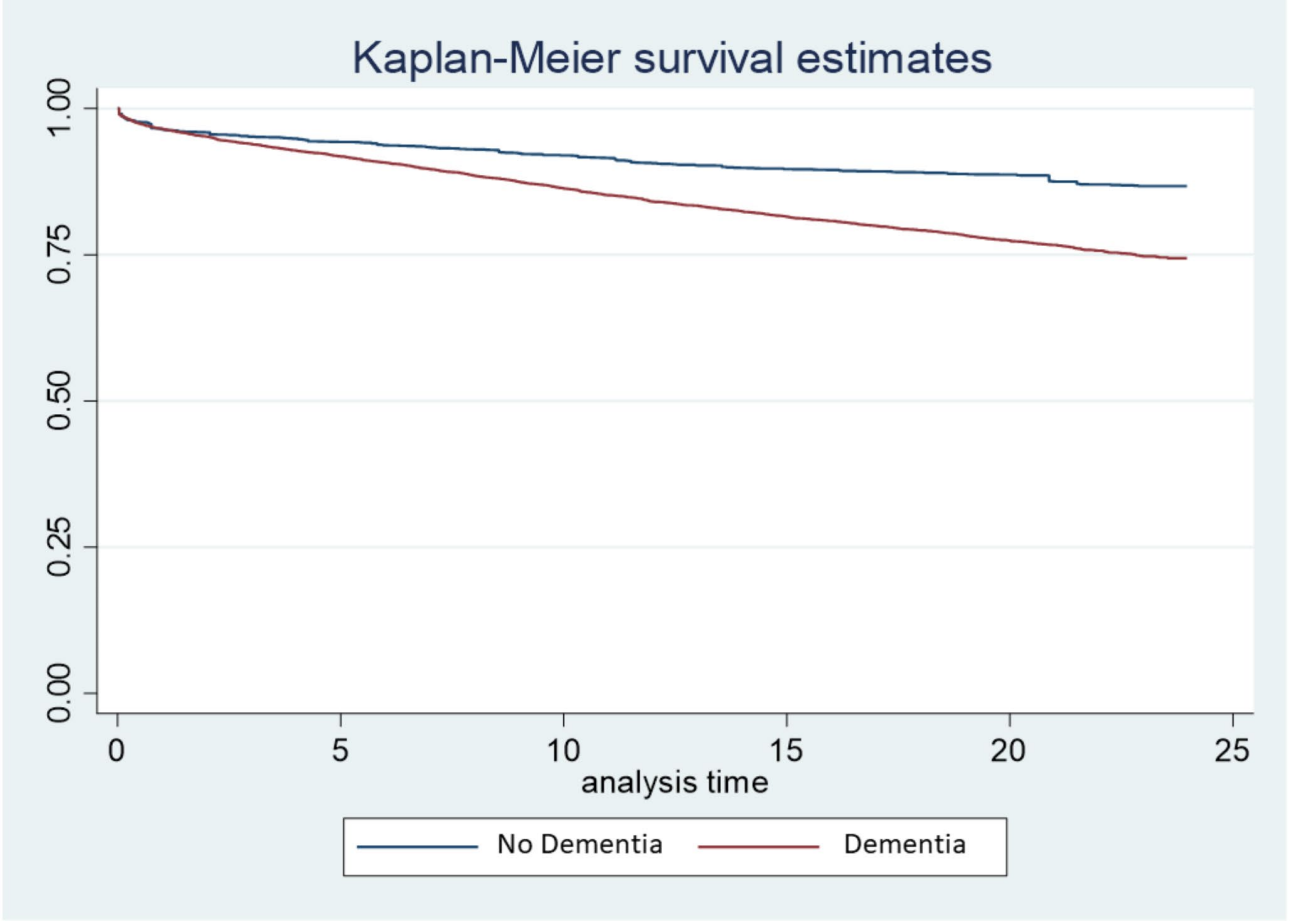
Three-year survival curves adjusted for confounding variables **Notes**: Adjusted for age, sex, Charlson comorbidity index and regions of Colombia by COX model

In descending order, other variables with a significant HR were age, chronic kidney disease, metastatic tumor, cancer, mild liver disease, and plegia, as indicated by the values adjusted by the Cox model and the hazards adjusted by PSM and Cox survival analysis (Fig. 2 Supplementary Table 3). Finally, age showed the strongest association with the decrease in survival rate, which significantly increased at > 50 years. Kaplan Meier curves at three years of post-hospitalization follow-up, unadjusted (Fig. 3) and adjusted for confounding variables (Fig. 4).

## Discussion

This study examines the impact of dementia on 1-year survival in hospitalized patients. Our findings reveal that dementia significantly reduces the survival rate of hospitalized patients, regardless of their comorbidities. This has important implications for the care of older patients with dementia, particularly those over 60, who face a substantial risk of decreased survival. Both the unadjusted HR and the HR adjusted by the Cox proportional hazards model and PSM demonstrated an initial overestimation of dementia risk, as indicated by the crude HR of 10.32 (95% CI 9.82 to 10.84). Following adjustment, the HRs were reduced to 1.69 (95% CI 1.60 to 1.78) and 1.32 (95% CI 1.02 to 1.71), respectively.

These findings are consistent with a 2019 study by Joling et al. [8], which used a cohort of 33,854 patients to estimate the time from dementia diagnosis to institutionalization and death. The study reported an average time from diagnosis to death of 5.0 years for patients with dementia and 9.6 years for those without, aligning with other studies reporting ranges from 1.1 to 11.7 years [22, 23]. The impact of dementia was quantified through the average between-group differences in survival time, revealing a decrease in life expectancy of approximately 1 month at 3-year intervals after adjustment using the Cox model and PSM. While few studies have documented this effect on survival, our findings provide valuable insights into the true influence of dementia on life expectancy [24–31].

In this study, the prevalence of dementia among hospitalized patients in the general population was found to be 1.04% (95% CI 1.03 to 1.47), with an increase to 5.18% (95% CI 5.16 to 5.19) among patients aged > 65 years. These figures were lower than those reported in previous studies [13, 32–35]; however, it is important to note that the prevalence in this study may be underestimated, potentially due to data underreporting.

Furthermore, age exhibited the most substantial correlation with increased mortality in patients with dementia, followed by the presence of certain comorbidities such as chronic kidney disease, cancer, the existence of a solid metastatic tumor, and plegia. The National Report of Vital Statistics of the United States in 2017 [36] reported an age-adjusted mortality rate for dementia at 66.7 deaths per 100,000 inhabitants. This underscores the significance of age as the most widely recognized risk factor [37, 38], as evidenced by the fact that 1.4% and 66.9% of deaths from dementia in 2017 occurred in patients aged < 65 years and ≥ 85 years, respectively. Consequently, older adults with dementia necessitate comprehensive and multidisciplinary management to address all their end-of-life requirements.

A significant strength of this study lies in the utilization of administrative data sourced from the Colombian Ministry of Health, covering approximately 45% of the population and ensuring a representative random sample, considering the health coverage percentage of 95.6% in 2016 [39]. The standardized coding across national databases further enhances the interpretability of these findings across the entire Colombian population. The incorporation of ICD-10 codes, CUPS, and prescribed medications for identifying comorbidities enables a more robust adjustment of confounding variables, as advocated by Schneeweiss et al. [40]. If these data are available for research purposes and maintain sufficient quality, such administrative data serves as a valuable alternative to costly prospective studies, significantly reducing the time required for data collection [8, 20]. Additionally, the potential for selection bias was minimized by extending the reported confirmed diagnoses in the databases two years before cohort establishment. Unlike previous studies in other regions, our approach avoids discrepancies in recording precise diagnoses across different dementia types in death certificates and clinical history [36]. Hence, an analysis based on dementia type was considered inappropriate. To the best of our knowledge, this study marks the first instance of utilizing administrative data in Colombia, facilitating the inclusion of a substantial number of hospitalized patients with dementia, revealing that 49.77% of the population received treatment.

Despite utilizing a highly standardized database, the absence of patient-specific clinical data limited our ability to assess disease severity. Additionally, the inability to differentiate between vascular and degenerative dementias represents a significant limitation, as these subtypes differ in causal factors, risk profiles, progression, and outcomes. The study also lacked information on patients’ social status, precluding the evaluation of factors such as attendance at chronic care units, caregiver presence, physical activity, and degree of dependency [27, 41].

### Limitations

Our findings indicated a low prevalence of dementia among hospitalized patients, considerably lower than that reported in the literature [3, 5, 6, 27, 41]. This finding can be explained by the fact that our population is based on hospitalized patients, introducing a bias related to the selection from a clinical setting. Unlike studies based on community samples, hospitalized patients may have a different prevalence of dementia, as not all cases of dementia are diagnosed or recorded in this context, and many patients may be hospitalized for reasons unrelated to neurodegenerative disease [6, 27, 41]. Additionally, this discrepancy may be due to a lack of data or incomplete data, leading to an underestimation of the true prevalence due to underreporting. Another notable limitation is the potential misclassification of dementia due to underdiagnosis or failure to recognize it in administrative records. It is possible that patients with undiagnosed dementia were incorrectly included in the cohort of patients without dementia, which could bias the study’s findings.

We acknowledge that the definition of incident cases used in our study has certain limitations. First, it relies exclusively on the data available in the administrative database, which may underestimate the prevalence of cases previously diagnosed in outpatient settings or outside the observation period. Additionally, underdiagnosis during early or asymptomatic stages of the disease, as well as the possibility that dementia was not the primary cause of hospitalization, could limit the accurate identification of incident cases.

Furthermore, the absence of data on the length of hospital stay is a critical limitation, as longer stays are well-documented among patients with dementia. Prolonged hospitalizations are associated with greater functional decline, increased dependency, and a heightened risk of hospital-acquired complications, such as infections, falls, fractures, delirium, and venous thromboembolism [3, 5, 7]. The lack of this information prevented a thorough assessment of its impact on survival outcomes.

Comparisons with people with dementia who were not hospitalized could provide additional insights into the relative impact of hospitalization on this population. However, the data available for this study did not include information on non-hospitalized individuals with dementia, limiting our ability to perform such analyses. Lastly, the association between survival and advanced-stage dementia diagnosis warrants further exploration, as advanced disease stages likely correlate with higher mortality due to complications.

Despite these limitations, our study benefits from a large, nationally representative sample covering diverse regions, ensuring significant external validity and reducing random error. This broad scope provides valuable insights into the survival outcomes of hospitalized patients with dementia in a unique healthcare context.

## Conclusions

Our findings reveal that dementia exerts a negative impact on the survival rate of hospitalized patients, irrespective of the various comorbidities they may be experiencing. Our investigation uncovered that dementia resulted in a reduction of 3-year survival by 0.98 months. This has significant implications for the care of older patients with dementia, particularly those aged above 60, as they face a substantial risk of reduced survival.

## Electronic supplementary material

Below is the link to the electronic supplementary material.

Supplementary Material 1

## Data Availability

No datasets were generated or analysed during the current study.
