# Supplementary material for: Survival outcomes among hospitalized patients with dementia: a propensity score matching analysis
Source: Acta Neurol Belg. 2025 Mar 15;125(3):771–81. doi: 10.1007/s13760-025-02746-7 (PMC12126351; doi:10.1007/s13760-025-02746-7)
Supplement: Supplementary file 1 — Supplementary Material 1 [file 13760_2025_2746_MOESM1_ESM.pdf]

Supplementary figure 1. Graphic representation of the study cohort

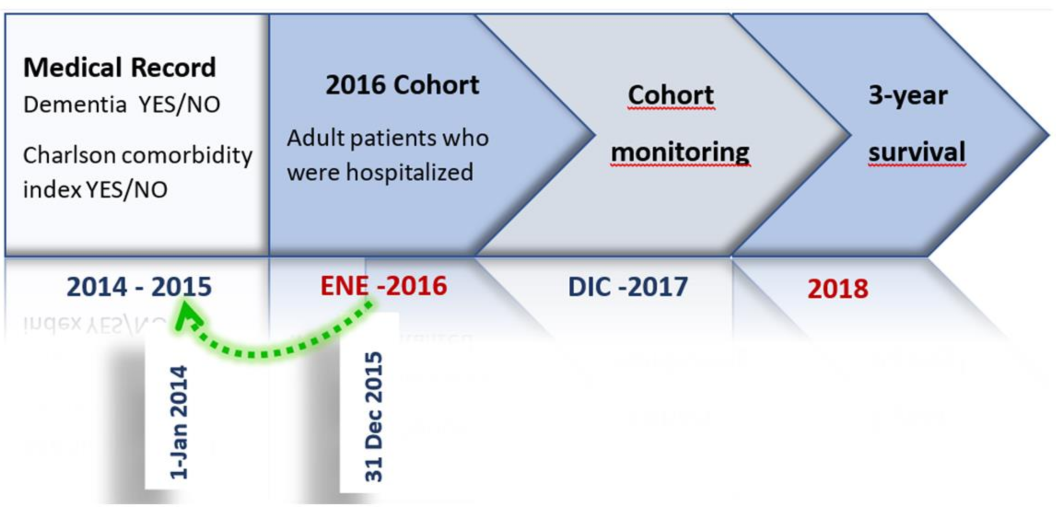

Supplementary figure 2. Directed acyclic graph model of 36-month survival in patients with dementia.

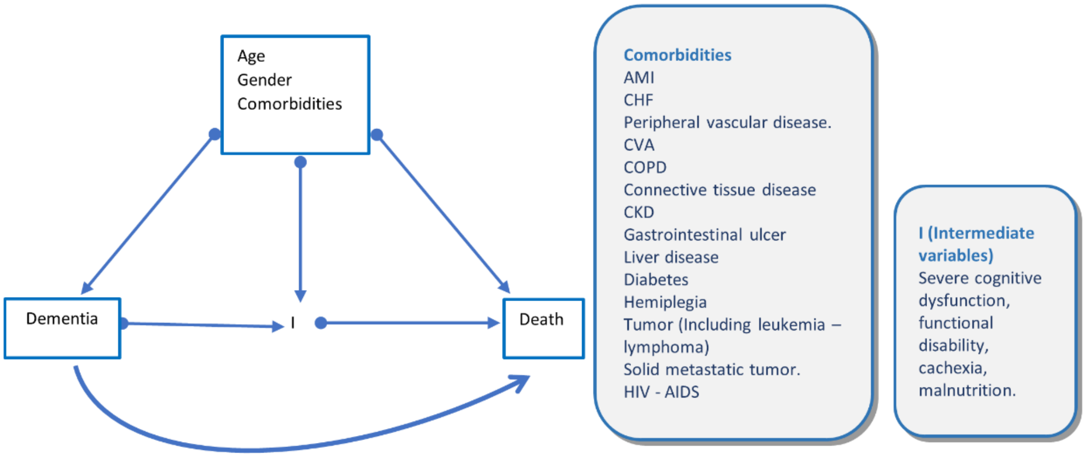

Notes: AMI, Acute myocardial infarction; CVA, cerebrovascular accident; COPD, Chronic pulmonary disease; CKD, Chronical Kidney Disease; CHF, Congestive heart failure; AIDS, acquired immunodeficiency syndrome.

Supplementary figure 3. Common support area for hospitalized patients with and without dementia

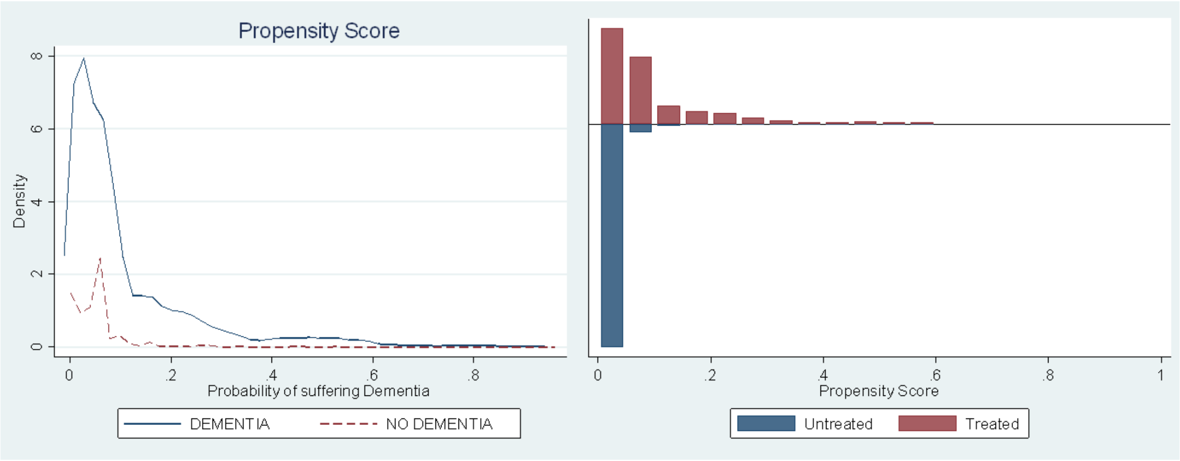

Supplementary figure 4. Unadjusted and adjusted Hazard ratio comparison by propensity score and proportional risk model in patients with dementia

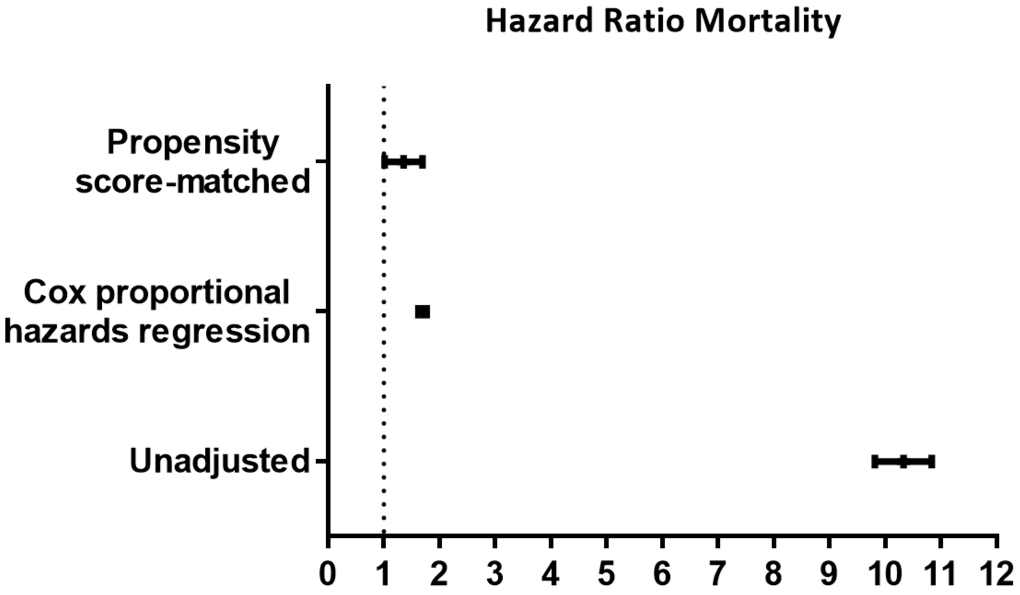

Supplementary table 1. Variables definitions

COMORBIDITY	ICD10 CODES	UNIQUE CODE OF HEALTH PROCEDURES AND Y MEDICATION
ACUTE MYOCARDIAL INFARCTION	I21* I22* I252 I255	360100 360101 360102 360102 360200 360201 360202
CONGESTIVE HEART FAILURE	I110 I130 I132 I50* I420 I425 I429 I43*	
PERIPHERAL VASCULAR DISEASE	K551 K558 K559 Z958 Z959 I70* I71* I720 I728 I739 I719 I731 I738 I771 I790 I792	380300 380910 383903 392204 392400 392501 392502 392503 392601 392602 392603 392604 392605 380300 392620
STROKE	G45* G46* I60* I69* I67* I681 I682 I688 I694 I698	395010 380101 380110 380210
DEMENTIA	F00* F01* F02* FO3* G30* G311 F03X F051 F010	*RIVASTIGMINA* *DONEPEZIL* *GALANTAMINA* *MEMANTINE* *DONEPEZIL MEMANTINE* *BUPROPION* *AMANTADINE*
CHRONIC OBSTRUCTIVE PULMONARY DISEASE	J441 I278 I279 J4* J60* J61* J62* J63* J64* J66* J67* J684 J701 J703 J448 J449 J410 J411 J42X J431 J432 J438 J439	*OXIGENO*
CONNECTIVE TISSUE DISEASE	M053 M058 M059 M060 M061 M063 M069 M050 M052 M051 M353 M320 M321 M328 M329 M353 M330 M331 M332 M339 M340 M341 M342 M348 M349 M353	*METOTREXATE* *SULFASALAZINA* *LEFLUNOMIDA* *PENICILAMINA* *RITUXIMAB* *ABATACEPT* *ETANERCEPT* *INFLIXIMAB* *ADALIMUMAB* *CERTOLIZUMAB* *GOLIMUMAB* *TOCILIZUMAB* *TOFACITINIB* *ANAKINRA* *TACROLIMUS* *ACTEMRA*

PEPTIC ULCER	K270 K271 K272 K273 K274 K275 K276 K279 K250 K251 K252 K253 K254 K255 K256 K257 K259 K260 K261 K262 K263 K264 K265 K266 K267 K269	S22224 441100 451301 451600 893904 901220 906022 906023 906024 438100 440100
MILD LIVER DISEASE	K709 K702 K703 K717 K740 K742 K746 K740 K742 K746 K743 K744 K745 K730 K731 K738 K739 k70* k73*	
DIABETES MELLITUS TYPE 2	E100 E101 E106 E108 E109 E110 E111 E116 E118 E119 E120 E121 E126 E128 E29 E130 E131 E136 E138 E139 E140 E141 E146 E148 E149 E140	*REPAGLINIDA* *NATEGLINIDA* *LIRAGLUTIDE* *EXENATIDE* *LIXIZENATIDE* *DULAGLUTIDE* *ACARBOSA* *MIGLITOL* *CLORPROPAMIDA* *TOLBUTAMIDA* *GLIBENCLAMIDA* *GLIMEPIRIDA* *GLICLAZIDA* *GLIBENS* *GLIPIZIDA* *INSULINA* *INSUMAN* *INSULEX* *INSULINA ASPART* *INSULINA GLULISINA* *INSULINA LISPRO* *INSULINA DETEMIR* *INSULINA GLARGINA* *INSULINA DEGLUDEC* *INSULINA NPH* *METFORMINA* *GEMIGLIPTINA* *EMPAGLIFOZINA* *FENFORMINA* *SITAGLIPTINA* *VILDAGLIPTINA* *LINAGLIPTINA* *ALOGLIPTINA*
PARAPLEGIA – HEMIPLEGIA	G81 G041 G820 G821 G822	
CHRONIC KIDNEY DISEASE	I120 I131 N03* N05* Z49* N18 N19 N25 N01 N074 N073 N072	389500 394300 399501 549002 392701 392702 394200 549001 549012 549800 549801 549802 S22220 S22223 549800

	N52 N19 N250 Z940 Z992	
DIABETES MELLITUS WITH COMPLICATIONS	E10* E11* E12* E13* E14* H360 H280 G590 G632 M142	
ANY TUMOR INCLUDING LEUKEMIA / LYMPHOMA (WITHOUT MALIGNANT NEOPLASIA OF THE SKIN)	C000 C001 C002 C003 C004 C005 C006 C007 C008 C009 C01X C020 C021 C022 C023 C024 C029 C030 C031 C039 C040 C041 C049 C050 C051 C052 C059 C060 C061 C062 C069 C07X C080 C081 C089 C090 C091 C099 C101 C102 C103 C104 C883 C887 C889 C900 C901 C91* C92* C93* C94* C95* C96*	
SEVERE LIVER DISEASE	K729 K766 K703 K767 K721	
METASTATIC SOLID TUMOR	C780 C7* C8* C781 C782 C783 C784 C785 C786 C787 C788 C79 C790 C791 C792 C793 C794 C795 C796 C797 C798	
AIDS	Z114 Z21X B200 B201 B202 B203 B204 B205 B206 B207 B208 B209 B210 B211 B212 B217 B219 B220 F028 R75X B220 B221 B24*	*ZIDOVUDINA* *LAMIVUDINA* *TENOFOVIR* *EMTRICITABINA* *DIDANOSINA* *NEVIRAPINA* *EFAVIRENZ* *ETRAVIRINA* *SAQUINAVIR* *LOPINAVIR* *ATAZANAVIR* *INDINAVIR* *NELFINAVIR* *RALTEGRAVIR* *ABACAVIR* *ABAMUNE* *RITONAVIR* *ATAZANAVIR*

Supplementary table 2. Sample size calculation

The sample size was calculated according to the number of deaths required in each of the exposed and unexposed groups; the formula is expressed as follows:
$(Z_{\beta} + Z_{1-\alpha})^2 / (P_A * P_B) * (Log_e^2 \Delta)$

Notes: Z_{β} : is the power; $Z_{1-\alpha}$: significance level; P_A : proportion of exposed patients; P_B : proportion of unexposed patients; Δ_0 : Hazard ratio = 1.5.

Supplementary table 3. Three-year mortality risk of hospitalized patients, COX risk model adjusted values.

Variable	Hazard Ratio (95% CI)	p value
**Dementia**	1.69 (1.60-1.78)	<0.001
**Gender**	1.48 (1.43-1.52)	<0.001
**Age**		<0.001
< 50	4.16 (3.90-4.44)	<0.001
50 - 59	9.33 (8.80-9.88)	<0.001
60 - 69	16.96 (16.04-17.92)	<0.001
70- 79	33.82 (32.00-35.73)	<0.001
80- 89	66.16 (61.94-70.67)	<0.001
90- 99	100.54 (74.98-134.80)	<0.001
**Comorbidity**		<0.001
CKD	3.45 (3.27-3.64)	<0.001
Diabetes mellitus	1.32 (1.24-1.41)	<0.001
CHF	1.39 (1.30-1.49)	<0.001
Peripheral vascular disease	1.11 (1.03-1.19)	0.006
Cerebrovascular disease	1.41 (1.34-1.48)	<0.001
COPD	1.29(1.25-1.34)	<0.001
Connective tissue disease	1.03 (0.97-1.10)	0.286
Peptic ulcer disease	1.01 (0.96-1.06)	0.567
Mild liver disease	2.13 (1.83-2.47)	<0.001
Metastatic solid tumor	2.48 (2.31-2.66)	<0.001
AIDS	1.40 (1.25-1.57)	<0.001
Diabetes mellitus with complications	0.90 (0.84-0.96)	0.002
Severe liver disease	1.50 (1.23-1.82)	<0.001
Acute myocardial infarction	0.93 (0.89-0.98)	0.015
Any tumor	2.45 (2.21-2.72)	<0.001
Plegia	1.51 (1.12-2.04)	0.006
**Region**		
Bogotá	1.15 (1.10-1.21)	<0.001
Central	1.26 (1.20-1.31)	<0.001
Oriental	1.16 (1.10-1.22)	<0.001
Pacific	1.07 (1.01-1.13)	0.008
Others state	1.07 (0.87-1.31)	0.476

Notes: CI, confidence interval; COPD, Chronic pulmonary disease; CKD, Chronical Kidney Disease; CHF, Congestive heart failure; AIDS, acquired immunodeficiency syndrome.
